# Neuroprotective Agents with Therapeutic Potential for COVID-19

**DOI:** 10.3390/biom13111585

**Published:** 2023-10-27

**Authors:** César A. Zaa, Clara Espitia, Karen L. Reyes-Barrera, Zhiqiang An, Marco A. Velasco-Velázquez

**Affiliations:** 1 School of Biological Sciences, Universidad Nacional Mayor de San Marcos (UNMSM), Lima 15081, Peru; cesar.zaa@unmsm.edu.pe; 2 Department of Immunology, Institute of Biomedical Research, Universidad Nacional Autónoma de México (UNAM), Mexico City 04510, Mexico; espitia@iibiomedicas.unam.mx (C.E.); karenreyesbarrera@iibiomedicas.unam.mx (K.L.R.-B.); 3 Texas Therapeutics Institute, Brown Foundation Institute of Molecular Medicine, University of Texas Health Science Center, Houston, TX 77030, USA; zhiqiang.an@uth.tmc.edu; 4 School of Medicine, Universidad Nacional Autónoma de México (UNAM), Mexico City 04510, Mexico

**Keywords:** COVID-19, SARS-CoV-2, neurological symptoms, neuroprotective compounds, flavonoids, terpenoids

## Abstract

COVID-19 patients can exhibit a wide range of clinical manifestations affecting various organs and systems. Neurological symptoms have been reported in COVID-19 patients, both during the acute phase of the illness and in cases of long-term COVID. Moderate symptoms include ageusia, anosmia, altered mental status, and cognitive impairment, and in more severe cases can manifest as ischemic cerebrovascular disease and encephalitis. In this narrative review, we delve into the reported neurological symptoms associated with COVID-19, as well as the underlying mechanisms contributing to them. These mechanisms include direct damage to neurons, inflammation, oxidative stress, and protein misfolding. We further investigate the potential of small molecules from natural products to offer neuroprotection in models of neurodegenerative diseases. Through our analysis, we discovered that flavonoids, alkaloids, terpenoids, and other natural compounds exhibit neuroprotective effects by modulating signaling pathways known to be impacted by COVID-19. Some of these compounds also directly target SARS-CoV-2 viral replication. Therefore, molecules of natural origin show promise as potential agents to prevent or mitigate nervous system damage in COVID-19 patients. Further research and the evaluation of different stages of the disease are warranted to explore their potential benefits.

## 1. Introduction

Patients presenting severe pneumonia by an unknown cause were reported in China in December 2019. By January 2020, Chinese authorities reported a novel coronavirus (CoV) as the cause of the illness [1,2]. This novel virus matched the lineage B of the genus betacoronavirus showing >85% identity with a bat SARS-like CoV [1]. Hence, this virus was included in the family containing Severe Acute Respiratory Syndrome (SARS) CoV (79% of genome identity), Middle East respiratory syndrome (MERS) CoV (50% of genome identity), and four other CoVs associated with the common cold [2,3]. Later, the virus was named SARS-CoV-2, and COVID-19 the disease caused by it [2,4]. By the end of January 2020, the World Health Organization (WHO) declared SARS-CoV-2 as a Public Health Emergency of international concern, and by March 2020, COVID-19 was declared a global pandemic [4]. As of 15 March 2023, more than 760 million people worldwide have been infected with SARS-CoV-2, with approximately 6.87 million deaths [5]. 

Since the beginning of the pandemic, different lineages of SARS-CoV-2 and multiple variants have emerged [6]. The appearance of variants with D614G mutation in the spike (S) protein quickly became dominant strains. This mutation allowed for better binding of the viral S protein to the angiotensin-converting enzyme 2 (ACE2) receptor, accelerating virus infectivity and spread [7]. To date, other variants with important clinical implications have appeared [8]. Such variants are classified according to the potential or known impact of the mutations on the effectiveness of health measures, the severity of the disease, and the ability to spread from person to person [5]. 

The emerging variants of SARS-CoV-2 are classified into variants of interest (VOI) and variants of concern (VOC). VOI have specific genetic markers associated with changes promoting increased virulence, reduced neutralization by antibodies generated by natural infection or vaccination, and the ability to evade detection or a decrease in the efficacy of vaccination. Eight VOIs have been described: Epsilon (B.1.427 and B.1.429); Zeta (P.2); Eta (B.1.525); Theta (P.3); Iota (B.1.526); Kappa (B.1.617.1); Lambda (C.37), and Mu (B.1.621) [9]. VOC are variants that meet the definition of VOI and that may be associated with one or more of the following: (1) increased transmissibility or detrimental change in the epidemiology of COVID-19; (2) change in the clinical presentation of the disease (more severe manifestations, including death); or (3) decreased effectiveness of public health and social measures [7]. The following VOCs have been described: Alpha (B.1.1.7); Beta (B.1.351); Gamma (P.1); Delta (B.1.617.2); Omicron (B.1.1.529) [9], with variants of the latter currently circulating [10].

Clinical manifestations of COVID-19 range from asymptomatic to mild respiratory tract infections and influenza-like illness to severe illness with lung injury, multiple organ failure, and death. Within such a wide clinical spectrum, multiple varieties of neurological symptoms have been reported. In the present narrative review, we provide a detailed description of the neurological symptomatology in COVID-19 patients and their incidence, compiled from original articles, meta-analyses, and systematic reviews. Furthermore, we discuss the literature generated in the previous four years regarding the mechanisms of SARS-CoV-2 nervous pathogenesis. Finally, we present in vitro, in vivo, and clinical evidence showing that neuroprotective compounds reduce the effects of COVID-19 in the nervous system by targeting the molecular pathways supporting SARS-CoV-2 infection and/or neuronal damage. 

## 2. SARS-CoV-2 Structure and Biology

SARS-CoV-2 is a single-stranded positive-sense RNA virus with an approximate 60–140 nm diameter with spike projections that emerge from the virions’ surface, a characteristic of the *Coronaviridae* family [1,11]. 

The initial 20 kb downstream the 5′ end of the viral genomic region is occupied by the open reading frame (ORF) 1a and 1b, which encodes the polyproteins (pp) 1a and 1ab that contain nonstructural proteins (NSPs). Polyprotein pp1a generates NSP1 to NSP11 whereas pp1ab comprises NSP1 to NSP16. Although NSPs are not included in the viral particle, they play an important role in RNA synthesis and processing, contributing to viral propagation [12]. The remaining 10 kb, preceding the 3′ end, code for four structural proteins (10): spike (S), membrane (M), envelope (E), and nucleocapsid (N) [13].

The S protein plays a key role in virus pathogenesis, infectivity, induction of immune response, and evolutionary mutation [14]. The receptor binding domain (RBD) of the S protein binds to ACE2, mediating SARS-CoV-2 entry to host cells [15,16]. Notably, protein S shows a high diversity, caused by selective pressures and adaptive changes over time, which determine a stabilizing interaction in the spike-ACE2 complex [17]. 

After binding, early viral entry via membrane fusion is promoted by S protein cleavage between S1 and S2 domains by the host protease TMPRSS2 (transmembrane protease serine 2) [18]. ACE2 and TMPRSS2 are expressed in multiple tissues, including lung, kidneys, small intestine, colon, brain, heart, liver, and blood vessels [19,20,21,22], making these tissues susceptible to viral infection. In cells expressing insufficient TMPRSS2, the ACE2-bound virus can be internalized via clathrin-mediated endocytosis in the late endolysosome, where the junction of S1–S2 subunits of the S protein is cleaved by endosomal proteases, especially cathepsin L [18,23]. After the S1 subunit is shed, the S2′ site is cleaved, either by TMPRSS2 on the surface cell or by cathepsins in endosomes, activating membrane fusion [18].

TMPRSS2 inhibitors and cathepsin inhibitors reduce virus infection with different efficacy, indicating that both entry routes are active and cooperate. Thus, the combined treatment with TMPRSS2 inhibitors and cathepsin inhibitors further reduces virus infectivity [15,24]. Accordingly, ACE2 [24], TMPRSS2 [25], and cathepsin L [26] are host-based targets for the development of anti-SARS-CoV-2 therapies.

After RNA release into the host cells, multiple NSPs control RNA transcription, translation, and protein synthesis required for viral replication; thus, such proteins have been targeted for therapeutic development. For example: (i) RNA-dependent RNA polymerase (RdRp, also known as Nsp12) is a crucial component of the genome replication/transcription complex of SARS-CoV-2; (ii) helicase (Nsp13) can unravel double-stranded (ds) DNA and RNA along the 5′–3′ direction and is vital to viral replication; and (iii) Mpro (also known as 3CLPro) and PLPro (papain-like protease) participate in the viral replication through proofreading and excision of the polyproteins [27]. 

Detailed reviews of SARS-CoV-2 structure and biology have been published recently [28,29].

## 3. SARS-CoV-2 Infection and Clinical Manifestations

SARS-CoV-2 affects homeostasis, which can lead to life-threatening systemic complications. In the critical SARS-CoV-2 infections, the routes of viral spread in blood vessels are lymphatic, hematogenous, direct invasion of adjacent tissues, and pathogenic implantation. SARS-CoV-2 has been detected in oral and nasal mucosa, stomach, heart, small intestine, colon, lymph nodes, blood samples, thymus, spleen, liver, kidney, and brain [30,31]. SARS-CoV-2, as SARS-CoV, causes pneumonia with severe lung damage in the worst cases, but SARS-CoV-2 is more transmissible due to a higher basic reproductive rate and drives more severe illness [32]. SARS-CoV-2 enters the lung from the mouth and throat [33]. Since the lung has a large surface area, it has increased susceptibility to inhaled viruses. In addition, its high vascularization allows the rapid spread of viral particles. 

Direct SARS-CoV-2 infection of lung cells has been described. For example, ACE2 is expressed in the type II alveolar epithelial cells [34], ciliated columnar epithelial [35], and AT2 (alveolar stem cells) [36]. These types of cells express multiple functional genes associated with the viral life cycle including TMPRSS2 [34,37]. SARS-CoV-2 infection of lung cells is accompanied by the infiltration of inflammatory cells, endothelial and inflammatory cell death, alteration of intracellular endothelial junctions, cellular swelling, and cell detachment from the basement membrane [38]. Therefore, pulmonary COVID-19 can be subdivided into four morphological stages, which include: (i) an early stage (day 0–1) with the presence of edema, initial epithelial damage and capillaritis/endothelialitis; (ii) diffuse alveolar damage (DAD) (days 1–7); (iii) the organizing phase (type II pneumocyte hyperplasia) (1 to several weeks); and iv) fibrotic stage of DAD (weeks to months) [39]. Detailed reviews of the pathogeny of severe pneumonia by SARS-CoV-2 infection were published previously [40,41,42,43]. 

As mentioned before, other organs/tissues can also be infected and damaged by SARS-CoV-2. For example, impaired kidney function has been reported in 13–14.4% of COVID-19 patients [44,45]. SARS-CoV-2 infects the kidney by binding to ACE2 in podocytes. Then, the virus could reach the apical membrane of the proximal tubule either by accessing the tubular fluid or during proximal tubule cell injury in patients with acute kidney injury (AKI) [46]. AKI in COVID-19 patients could be mediated by innate immune system over-activation, cytokine release, complement activation, angiotensin II (Ang II) hyperactivity, development of a hypercoagulable state, hypovolemia secondary to excessive diuresis, and/or increased venous pressure secondary to high positive pressure at the end of expiration [45,47].

A detailed review of the SARS-CoV-2 target organs and the local pathogenesis mechanisms are available in [48].

### COVID-19 Neurological Symptoms

**Figure 1:**
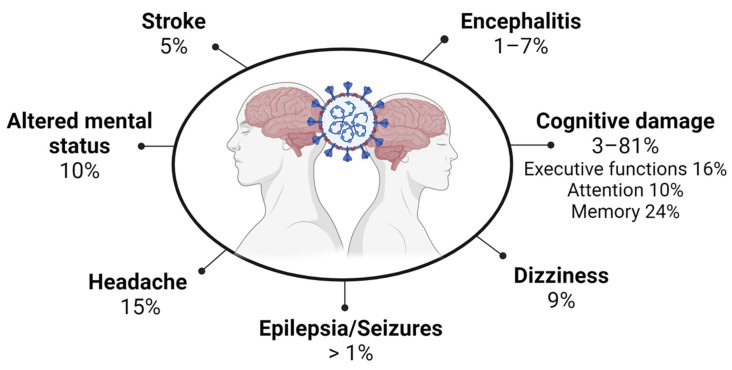
Neurological symptoms caused by SARS-CoV-2 infection and their reported frequencies in COVID-19 patients. Created with Biorender.Com.

A comprehensive exploration of neurological disorders conducted by specialists from the World Federation of Neurology reports that SARS-CoV-2 infection affects the central nervous system (CNS), the peripheral nervous system (PNS), and skeletal muscle [49]. Neurological symptoms in COVID-19 patients (Figure 1), which are more frequent in severely ill patients (45.5%) [50], include:

- Taste and olfactory dysfunctions (ageusia/anosmia). These are the most common PNS neurological symptoms of COVID-19. They develop in the early stages of the disease and can precede most symptoms; thus, they are considered useful diagnostic markers [51].
- Headache: Headache is the most common nonspecific neurological symptom, with an estimated combined prevalence of 14.7% [52].
- Altered mental status/confusion/delirium: Acute confusion/delirium may be a primary manifestation and the only presenting symptom of COVID-19 without evident lung disease [53]. The combined prevalence of altered consciousness/altered mental status is around 9.6% [52].
- Dizziness: Its combined prevalence in patients with COVID-19 is 8.77%, according to a systematic review [54].
- Stroke: The prevalence of acute ischemic cerebrovascular disease in hospitalized COVID-19 patients with severe infections reaches approximately 6% [50]. In a retrospective study of 221 patients, 11 (5%) had ischemic stroke, one (0.5%) had cerebral venous thrombosis, and one (0.5%) showed cerebral hemorrhage [55]. Elderly patients with COVID-19 either with vascular risk factors or concomitant diseases such as hypertension or diabetes mellitus are at increased risk of developing cerebrovascular complications [55,56,57]. COVID-19 patients with acute ischemic stroke report visual deficits including hemianopia [58].
- Epilepsies and seizures: COVID-19 lowers the seizure threshold in patients with existing seizure disorder and may also worsen a controlled condition [59]. SARS-CoV-2-associated seizures can also occur because of meningitis/encephalitis [60]. Several studies have reported that the incidence of acute symptomatic seizures due to COVID-19 is low compared to SARS or MERS. Two large studies each with >4000 COVID-19 patients from Iran or New York reported an incidence of <1% [61]. However, the prevalence might be higher in COVID-19 patients with preexisting or other comorbidities [61,62].
- Encephalitis: A systematic meta-analysis study found that the incidence of encephalitis as a complication of COVID-19 is <1% for all patients but rises to 6.7% in those with severe disease. In addition, the mortality rate of patients with encephalitis as a complication of COVID-19 is 13.4%, almost four times that of the general population of COVID-19 patients [63].
- Guillain-Barré syndrome (GBS): COVID-19 patients with GBS can present weakness and paraesthesia of the lower extremities, progressing over several days and that can lead to generalized tetraparesis or tetraplegia [64]. Most of these patients have a demyelinating electrophysiological subtype corresponding to acute inflammatory demyelinating polyneuropathy [65]. In addition, acute motor axonal neuropathy and acute motor and sensory axonal neuropathy have been reported in COVID-19 patients [66,67].
- Cognitive damage: COVID-19 can cause a cognitive deficit, mainly in attention and executive function, and verbal learning; and the incidence is associated with the severity of COVID-19 [68,69]. The prevalence of cognitive impairment due to COVID-19 infection is not well determined. Reported studies have been limited by sample sizes or suboptimal measures of cognitive functioning [70]. Some of the post-COVID cognitive symptoms may be associated with other systemic symptoms [37]. However, systematic reviews analyzing COVID-19 patients show that cognitive impairment ranges from 2.6% to 81% before or at 12 weeks of infection. After 12 weeks, cognitive decline ranged from 21% to 65% [34,71]. Another meta-analysis study that included 27 studies with 2049 individuals found impairment in executive functions (16%), attention (10%), and memory (24%) in post-COVID-19 patients [70,72]. Consistent results have been reported, even in recovered COVID-19 patients, showing lower overall cognition compared to healthy controls up to 7 months post-infection [72].
- Impaired cranial nerves: Cranial nerve symptoms are more frequent and severe in COVID-19 than in previous SARS and MERS outbreaks, suggesting that SARS-CoV-2 has a more neurotrophic and aggressive neuroinvasion. Multiple cranial nerve abnormalities in COVID-19 patients have been reported [73]. Although most olfactory sensory neurons (OSNs) do not express ACE2 and TMPRSS2 [74,75], there is evidence that sustentacular cells can serve as a vehicle for the virus, through transcytosis or exosomes, to infect OSNs and reach the brain. Moreover, the virus can impair the OSN renewal process by sustentacular cells in the olfactory epithelium or cause direct damage to CNS neurons. Another possible mechanism is that stem cells in the olfactory epithelium expressing ACE2 are infected with virus from sustentacular cells, and when these cells mature in OSNs they can carry SARS-CoV-2 to the CNS [74,76].

Since the prevalence of hyposmia/anosmia (27.2%) and hypogeusia/ageusia (30.8%) is high in several studies, it is likely that the most frequently involved cranial nerves are I, VII, IX, and X [77]. However, if these common symptoms are produced by virus damage to the CNS or systemic immune response, it can be expected that other cranial nerves are affected [78]. 

In addition, it is known that the neurons of the terminal nerve (cranial nerve “0”) enter the brain through the cribriform plate and connect the nasal epithelium with the brain centers’ caudal to the olfactory bulb, the medial forebrain (septum), the preoptic area, and the hypothalamus. Although they are rare in humans (in contrast to the greater numbers found in marine mammals), they innervate blood vessels and are in direct contact with the subarachnoid space. These characteristics make it an almost ideal conduit for the transmission of SARS-CoV-2 to the caudal centers of the brain, cerebrospinal fluid, and the vascular system [76].

- Skeletal muscle symptoms: COVID-19 patients present fatigue, myositis, myalgia, and skeletal muscle injury. Most coronavirus infections can cause functional defects and myalgia or generalized weakness in skeletal muscles with elevated levels of creatine kinase [79]. In SARS-CoV-2-positive individuals, several cases report skeletal muscle symptoms, including back pain, dyskinesia, and lower limb paresthesia [80]. Myalgia prevalence varies widely between studies, from 3.36% to over 64%, with an estimated combined prevalence of around 19.3% [52].

An investigation of 213 COVID-19 cases indicated that 85.2% of patients had significantly elevated serum creatine kinase [81], which can be caused by skeletal muscle injury [79]. Likewise, cases of rhabdomyolysis have been described [82].

## 4. Mechanisms of SARS-CoV-2-Induced Neurological Damage 

Both direct or indirect effects of SARS-CoV-2 on the central nervous system can contribute to neurological and/or neuropsychiatric symptoms of COVID-19 [83]. In the following sections we review the underlying mechanisms.

### 4.1. Direct Neuronal Damage

SARS-CoV-2 can reach the CNS via the olfactory tract [84]. The human olfactory epithelium is a pseudostratified epithelium composed of Bowman’s glands, horizontal and globose basal cells, microvillar cells, sustentacular cells, and olfactory sensory neurons that extends a single axon towards the olfactory bulb in the brain. Sustentacular cells in the olfactory epithelium exhibit high levels of ACE2 and TMPRSS2 [75] and a study in hamsters showed SARS-CoV-2 active infection in those cells [85]. In agreement, autopsies of COVID-19 patients revealed that the olfactory sensory epithelium was severely damaged [75]. Despite the absence of ACE2 and TMPRSS2 in the olfactory sensory epithelium, the olfactory nerve tissue was found to be positive to SARS-CoV-2 in post-mortem examinations of COVID-19 patients [86]. Thus, it is possible that the replication of virions in the olfactory epithelium leads to infection in the olfactory bulb as blood vessels and pericytes in this brain region express both proteins [87,88]. These neurotrophic properties of SARS-CoV-2 explain the onset of anosmia as a prior symptom [89]. Interestingly, in mice, ACE2 and TMPRSS2 expression in the olfactory epithelium increases with age [75,90], suggesting a possible mechanism by which older patients are more vulnerable to the disease and neurological complications. 

Furthermore, the virus can reach brain tissue by the hematogenous route, in which endothelial cells or leukocytes are infected by the virus that passes from the bloodstream to the CNS [75] across the blood–brain barrier (BBB) [91,92] or by transmigration of peripheral immune cells, following the “Trojan horse” mechanism [93,94]. For example, brain vascular cells and choroidal barrier cells robustly express several genes that are relevant for SARS-CoV-2 entry into the brain [95]. SARS-CoV-2 infects and crosses an in vitro model of the BBB comprising primary brain microvascular endothelial cells and astrocytes [96]. Infection of ACE2-overexpressing primary human endothelial cells by SARS-CoV-2 induces the overexpression of coagulation factors, adhesion molecules, and pro-inflammatory cytokines, as well as the formation of multinucleated syncytia and endothelial cell lysis [97]. Consequently, SARS-CoV-2 alters the function and integrity of the BBB, which contributes to viral encephalopathy [98,99].

Furthermore, ACE2 receptors have been found in glial cells of the brain and spinal neurons, so SARS-CoV-2 can adhere, multiply, and cause direct damage to neuronal tissue [100]. Neuronal infection has been associated with neurodegeneration and neurovascular remodeling [101], causing cerebral vascular/endothelial dysfunctions that can generate cerebral circulatory disturbances [102]. Helms et al., using perfusion imaging, demonstrated in patients with COVID-19 that SARS-CoV-2 neuroinvasion causes bilateral frontotemporal hypoperfusion, demonstrating cerebral circulatory impairment [103]. As consequences of cerebral hypoxia, COVID patients can show cerebral vasodilation, brain cell swelling, interstitial edema, obstruction of cerebral blood flow, and even headache due to ischemia and congestion [104].

### 4.2. Indirect Effects

Exacerbated inflammation participates in the damage to nervous tissue, as in other target organs. SARS-CoV-2 elicits an exacerbated and deregulated immune response of soluble immune mediators, termed a “cytokine storm” [105]. Multiple immune mediators, such as IL-1β, IL-6, CXCL10, TNFα, and other diverse cytokines are produced in response to SARS-CoV-2 infection and have been associated with functional alterations or tissue damage in different organs, including the brain [106]. 

In addition, elevated levels of pro-inflammatory cytokines could participate in aggravating neuropathies during critical COVID-19 illness. The overproduction of systemic inflammatory factors (cytokines, nitric oxide, and oxygen radicals) has been associated with the malfunction of peripheral nerves [107] as well as microvascular disorders and electrical and metabolic (channel) disturbances in muscle cells [108].

In addition, chronic damage to other systems can also damage the CNS through ischemia, metabolic dysfunction, and hormonal dysregulation [109]. Coagulopathy and endotheliopathy triggered by cytokine storms are potential mechanisms causing ischemic stroke in COVID-19 patients [110,111]. Furthermore, COVID-19 patients have elevated levels of von Willebrand factor (VWF) antigen, VWF activity, and factor VIII [112], leukocytosis, thrombocytopenia, increased partial thromboplastin time, and low levels of antithrombin activity [113]. COVID-19 patients are at an increased risk of developing venous thromboembolism and disseminated intravascular coagulation [114].

Cerebral venous sinus thrombosis (CVT) can be caused by the hypercoagulable state in SARS-CoV-2 infection, which may be triggered by endothelial dysfunction that predisposes vessels to thrombus formation, platelet dysfunction, hypoxia, and/or alterations of the complement system [115,116]. CVT may cause generalized neurological deficits [117] and there are multiple reports of its association with SARS-CoV-2 infection [118,119].

Moreover, the renin-angiotensin-aldosterone system (RAAS) can contribute to the appearance of brain damage and systemic hyperinflammatory state in COVID-19 patients [120,121]. It has been reported that during SARS-CoV-2 infection: (1) the local levels of angiotensin II (Ang II) increase, acting on angiotensin II type 1 receptors (AT1), and thus increasing arterial pressure; (2) there is endothelial dysfunction in the cerebral vessels in the CNS, which increases the risk of cerebral hemorrhage; and (3) the generation of Ang (1–7) decreases, preventing the vasodilator, neuroprotective, and antifibrotic effects of Ang (1–7)/Mas receptor signaling [122,123].

### 4.3. Oxidative Stress

An overproduction of reactive oxygen species (ROS) and the deprivation of antioxidant mechanisms are known to be crucial for viral replication and subsequent virus-associated disease, as shown by increased ROS levels and impaired antioxidant defense during SARS-CoV-2 infection [124]. The viral protease Mpro activates nuclear factor kappa B (NF-kB)-mediated transcription, which correlates with increased levels of intracellular ROS [125]. In addition, Mpro causes a significant increase in ROS production in HL-CZ cells, which, in turn, induces cellular apoptosis. Similarly, SARS-CoV-2 increases oxidative stress in nervous tissue, which contributes to neuronal cell death [126,127]. A post-mortem case study showed that 37 of 43 COVID-19 patients had astrogliosis and 34 had microglial activation in the brainstem and cerebellum [128]. In a preclinical trial, neuronal microgliosis in the brain has been observed to persist beyond SARS-CoV-2 clearance [129].

### 4.4. Protein Misfolding

Protein misfolding and aggregation have also been reported in COVID-19. Interactions between the S protein of SARS-CoV-2 and its receptor ACE2 favor the spread of cytosolic prions and tau aggregates [130]. 

The RBD domain of the S1 subunit from SARS-CoV-2 S protein (RBD SARS-CoV-2 S1) binds heparin and heparin-binding proteins, accelerating the pathological aggregation of brain proteins, including Aβ (amyloid beta), α-synuclein, tau, prion, and TDP-43 RRM [131]. In addition, SARS-CoV-2-infected hamsters develop microgliosis in the olfactory bulb and selective accumulation of hyperphosphorylated tau and α-synuclein in the cortex after virus clearance, indicating that proteinopathies can be generated in neurons post-infection [129]. Although further studies are required, this evidence suggests that protein misfolding may play a role in the neurological symptoms caused by SARS-CoV-2 infection. 

### 4.5. Changes in Neurotrophins Expression

Neurotrophins are growth factors acting as regulators of neuronal survival, development, function, and plasticity [132]. Neurotrophins include nerve growth factor (NGF), brain-derived neurotrophic factor (BDNF), neurotrophin-3 (NT-3), and neurotrophin-4 (NT-4) [133]. In addition to their classical functions, they regulate axonal and dendritic growth and guidance, synaptic structure and connections, neurotransmitter release, and long-term potentiation, a cellular mechanism underlying memory and learning [134]. The circulating levels of BDNF [135] and NGF [136] are reduced in adult COVID-19 patients compared to healthy individuals. BDNF reduction is higher in patients > 60 years of age [137], indicating age-dependent effects. Reductions in serum BDNF correlate with the severity of the disease [137,138] and cognitive impairment after recovery [139]. Interestingly, adult COVID-19 patients that required supplemental oxygen had even lower BDNF serum concentrations [135], showing an interplay between deregulated BDNF levels and viral hypoxia. These findings support the role of neurotrophins in regulating neurological outcomes in COVID-19 patients. However, further studies are required to define the extent of their participation and the mechanisms involved, especially in the long-lasting effects of this disease.

## 5. Neurodegenerative Diseases and COVID-19 Share Mechanisms of Neural Dysfunction

**Figure 2:**
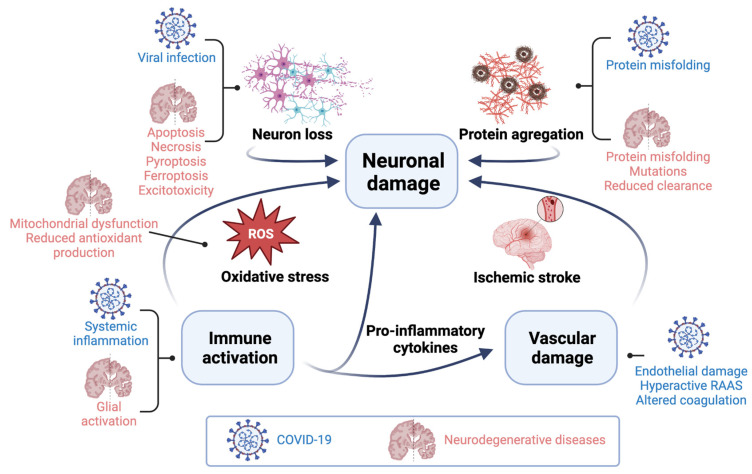
COVID-19 and neurodegenerative diseases share common mechanisms of neuronal damage. RAAS: Renin-angiotensin-aldosterone system. Created with BioRender.com.

Neurodegenerative diseases are triggered by a combination of genetic, epigenetic, and environmental factors [140]. These diseases share mechanisms of neural damage with COVID-19 described above (Figure 2). Since such mechanisms have been studied for several decades, they may provide a starting point for the identification of specific neuroprotective agents with COVID-19 application.

For example, oxidative stress, the formation of free radicals, and/or a dysfunction of the antioxidant system causes brain damage due to its high oxygen demand and its abundance of lipid cells susceptible to peroxidation [141]. This, in turn, induces altered intracellular signaling that can lead to a dysregulation of the inflammatory response [142] and/or cellular damage by altering the mitochondrial oxidative phosphorylation [143]. Thus, reducing ROS generation, lipid peroxidation, DNA damage, and protein oxidation, and modifying the release of mitochondrial factors are desirable goals in the therapeutic intervention of neurodegenerative diseases [144,145].

Similarly, neuroinflammation is a feature of neurodegenerative diseases. Innate immune cells within the CNS (microglia and astrocytes) as well as infiltrating immune cells are chronically activated in multiple sclerosis (MS) [146]. The accumulation of inflammatory cells and soluble mediators sensitizes neurons to further insults, triggering neurodegeneration by inducing apoptosis, necroptosis, neuronal autophagy, retrograde degeneration, and demyelination [147]. Accordingly, altering the concentration of chemokines and inflammatory cytokines, as well as the activation of astrocytes and microglia have been pointed to as goals of therapies in neurodegenerative diseases [144,145].

The death of neuronal cells is an important mechanism of neurodegenerative pathogenesis and is associated with alterations in the signaling cascades of cell death such as apoptosis, necroptosis, pyroptosis, ferroptosis, and autophagy-associated cell death. Aberrant activation of cell death pathways results in an unwanted loss of neuronal cells and function [148]. These processes can be triggered by intracellular or extracellular stimuli and inflammatory processes [149].

Excitotoxicity of neuronal cells (cell death due to excessive exposure to glutamate or overstimulation of NMDA glutamatergic receptors), is also common in neurodegenerative diseases [150]. Because of that, therapeutic goals include the modification of caspase activation and expression of proapoptotic proteins [144,145], and reduction in excitotoxic [151].

Finally, aberrant protein misfolding, aggregation, and accumulation are hallmarks and pathological features of neurodegenerative diseases such as prion diseases, Alzheimer’s disease (AD), and Parkinson’s disease (PD) (see [152] for a review). Reducing the formation of dysfunctional proteins caused by misfolding and agglomeration is a desirable effect in those neurodegenerative diseases [144,145].

However, only a few drugs have shown efficacy in neurodegenerative diseases and have clinical application. For example, AD is treated with acetylcholinesterase inhibitors (donepezil, galantamine, and rivastigmine), which improves cholinergic neurotransmission [153], and/or the N-methyl-D-aspartate (NMDA) receptor antagonist memantine [154], which prevents excessive continuous activation of extrasynaptic NMDA receptors, reducing excitotoxicity [155]. Thus, new potential pharmacological agents, with these or other relevant activities, are currently being sought [154].

## 6. Natural Products with Reported Neuroprotective Effects Could Reduce COVID-19 Neurological Symptoms

Evidence suggests that a healthy lifestyle that includes a balanced diet rich in bioactive compounds reduces the risk of developing CNS pathologies [156]. Multiple compounds from natural sources, mainly from medicinal plants, have been identified as modifiers of the pathogenic causes for various neurodegenerative disorders [157,158,159]. 

In this section we review the use of bioactive natural compounds as neuroprotective agents and discuss the evidence that suggests that they may be useful in COVID-19 treatment. Our literature search identified natural compounds with evidence of neuroprotection from common chemical classes, mainly flavonoids, alkaloids, and terpenoids. Furthermore, some of those compounds with neuroprotective activity may elicit direct effects on the SARS-CoV-2 viral cycle, as reported for multiple compounds from natural sources [160,161,162], making them more attractive candidates for adjuvant therapies. 

### 6.1. Flavonoids

Flavonoids are an important group of polyphenols with a wide range of biological activities. Flavonoids have been shown to be particularly effective in blocking pathological pathways associated with aging and neurodegeneration [163]. Bioactive flavonoids have good bioavailability and stability in circulating plasma and many of them reach the CNS [164,165,166], exerting their protective/restorative capacities in different neuronal populations.

Flavonoids can elicit two different effects that may be beneficial in COVID-19 patients: neuroprotection, mainly because of their antioxidant activity, and modulation of inflammation. For example, the flavone apigenin reduces AD-associated memory impairment, prevents oxidative stress, decreases Aβ plaque load [167], inhibits inflammatory stress, limits apoptotic cell death, and reduces neuronal hyperexcitability [96,168,169]. Furthermore, apigenin attenuates microglial activation and neuroinflammation, counteracting dopaminergic neuronal loss, improving locomotor ability in a PD model [170,171].

Quercetin, a flavonoid ubiquitous in fruits and medicinal herbs, has been widely studied for its neuroprotective effects. It antagonizes neuronal toxicity due to oxidative stress, suppresses neuroinflammation by down-regulating the activation of proinflammatory pathways mediated by NF-kB and iNOS, stimulates neuronal regeneration, and inhibits Aβ aggregation and tau phosphorylation [172]. Epicatechin is a flavonol found in blueberries, tea, cocoa, and grapes. It crosses the BBB [173] and exhibits neuroprotection through anti-apoptosis and anti-mitophagy effects in a model of Parkinson’s disease (PD) [174]. The combination of quercetin and epicatechin synergistically reduces ischemic neuronal cell death, preserves mitochondrial respiratory capacity, and confers protection against hypoxic-ischemic brain damage [175].

Hesperidin and neohesperidin, flavonoids present in citrus, enhance the content of glutathione (GSH) and the antioxidant enzymes catalase (CAT) and superoxide dismutase (SOD) in animal models of ischemic stroke [176], and activates the Akt/Nrf2/HO-1 signaling pathway to inhibit oxidative stress and protect brain damage induced by cerebral artery occlusion [177].

**Figure 3:**
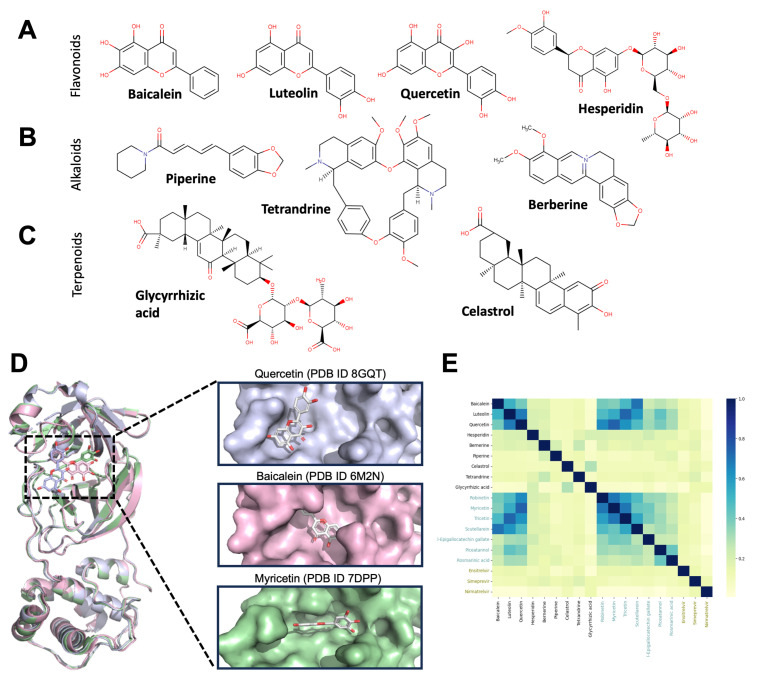
Structure of neuroprotective compounds and comparison with SARS-CoV-2 Mpro inhibitors. (**A**–**C**) Chemical structure of the flavonoids (**A**), alkaloids (**B)** and terpenoids (**C**) listed in Table 1. (**D**) Ribbon representation of SARS-CoV-2 Main protease (Mpro) crystal structures with the flavonoids quercetin, baicalein, or myricetin bound to the catalytic site (insets). (**E**) Structural comparison by extended connectivity fingerprints of compounds from Table 1 and reported Mpro inhibitors. Compounds in blue are natural products with an Mpro IC50 < 10 μM [160], whereas compounds in green are Mpro inhibitors that have reached clinical use.

Furthermore, we identified multiple flavonoids that display a direct anti-SARS-CoV-2 effect (Figure 3A and Table 1), which may be further beneficial for COVID-19 patients. The direct antiviral activity of flavonoids is not new and has been extensively reported [178,179,180]. The anti-SARS-CoV-2 activity of flavonoids was proposed at the beginning of the pandemic by computational experiments using SARS-CoV-2 Mpro as a target and confirmed later by crystallography (Figure 3D). Quercetin, baicalein, and luteolin show anti-SARS-CoV-2 activity and share structural features with reported Mpro inhibitors from natural sources (Figure 3E), suggesting they target viral proteins in addition to the neuroprotective actions. Subsequent preclinical evaluations confirmed a direct inhibition of viral entry and/or replication, as well as attenuation of systemic inflammation. 

Luteolin is a flavone widely distributed in the plant kingdom. Luteolin protects hippocampal damage and prevents learning defects in a rat model of AD [181]. Interestingly, luteolin attenuates microglial activation and excessive production of TNF-α, nitric oxide, and superoxide [182] and can reduce the activation of the TRIF-dependent signaling pathway of Toll-like receptors [183]. In addition, in SHSY human neuronal cells, it inhibits the transcription of β-secretase 1 (BACE-1), responsible for generating Aβ peptides [184]. It also reversibly inhibits human butyrylcholinesterase [185], which is one of the neuroprotective strategies of approved drugs. Luteolin has been shown to be an allosteric modulator of the S protein of SARS-CoV-2 [186] and inhibitor of Mpro [187]. 

Kaempferol is a flavonol present in various vegetables such as green tea with reported antiviral, immunomodulatory, and antioxidant effects [188,189]. Kaempferol can overcome the blood–brain barrier (BBB) with a single dose, reaching the hippocampus, frontal cortex, striatum, and cerebellum [190]. It has shown in vivo neuroprotective activity by attenuating the activation of the TLR4/NF-κB pathway in LPS-activated microglial cells [191]. Kaempferol modulates the antiepileptic target synaptic vesicle transporter 2A (SV2A) [192], inhibits 5-HT 3A receptors [193] involved in memory and cognitive functions, and blocks acetylcholinesterase (AChE) [194] implicated in cognitive dysfunction and memory loss associated with AD. Kaempferol decreases epileptic seizures in a rat model of chronic epilepsy, comparable to the control antiepileptic drug [192]. Kaempferol interacts with active sites of RdRp and Mpro from SARS-CoV-2 [195,196].

### 6.2. Alkaloids

Several alkaloids alter the pathophysiology of AD by functioning as muscarinic receptor agonists, antioxidants, acetylcholinesterase and butyrylcholinesterase inhibitors, α-synuclein aggregation inhibitors, anti-amyloid, and monoamine oxidase (MAO) inhibitors [197]. For example, galantamine is an AChE inhibitor that improves cholinergic neurotransmission, which is impaired in AD [198]. Piperine, a major alkaloid found in long pepper (*Piper longum*), showed efficacy in attenuating oxidative stress and improving cognition in the rat model of AD [199]. In the 6-hydroxydopamine (6-OHDA)-induced parkinsonian rat model, it decreased the inflammatory markers IL-1β and TNF-α [200]. In epilepsy, piperine delays tonic-clonic seizures by raising the cortical and hippocampal level of serotonin and GABA [201]. Others, such as harmaline, were shown to offset the toxic effects of dopamine oxidation in brain mitochondria, and together with harmine, increased antioxidant enzymes such as SOD and GSH peroxidase [202]. 

As for flavonoids, we identified alkaloids that, in addition to a neuroprotective effect, may display anti-SARS-CoV-2 activity (Figure 3B and Table 1). Berberine is an isoquinoline present in *Tinospora cordifolia* and roots, rhizomes, and stem bark of several medicinal plants of the Ranunculaceae, Rutaceae, and Berberidaceae families [203]. Berberine effectively crosses the BBB, which allows it to elicit neurotrophic and neuroprotective effects [204]. These effects are associated not only with its antioxidant action, but with the modulation of enzymes, neurotransmitters and molecular targets involved in neuropathology [205,206,207,208,209,210]. For example, berberine directly reduces ROS [211,212] and activates antioxidant mechanisms by regulating key signaling pathways, such as the P13K/AKT/Bcl-2 pathway and the Nrf2/HO-1 pathway [213]. Berberine is reported as an inhibitor of BACE1 and prevents Aβ 1–42 aggregation to delay the pathological process in AD [214,215,216]. It also improves motor stability and reduces dopaminergic neuron loss in PD [217] and reduces the deposition and aggregation of mutant huntingtin in HD, improving the coordination of movement and motor function [218]. 

Berberine reduces SARS-CoV-2 infectivity and blocks SARS-CoV-2 replication through direct interaction with the virion in Vero E6 cells and in human nasal epithelial cells [219,220]. In silico studies indicate that it may inhibit the function of SARS-CoV-2 Mpro [221,222]. Through molecular docking and network pharmacology, it was found that berberine inhibits pulmonary fibrosis in COVID-19 pneumonia by reducing TNF-α, IL-6, STAT3, and CCL2 [223]. Likewise, berberine/NIT-X nanoparticles inhibited the replication of SARS-CoV-2, the expression of the ACE2 and TMPRSS2 genes in the human lung epithelial cell line infected with SARS-CoV-2, and the expression of inflammatory cytokines and chemokines [81]. In a clinical study, berberine reduced circulating inflammatory mediators in patients with severe COVID-19 [96].

### 6.3. Terpenoids

The monoterpenoid carvacrol exhibits neuroprotective activities against cerebral infarction and the associated neurological deficits [224]. Those effects can be mediated by the reduction in inflammation caused by NF-κB inhibition, and inhibition of apoptosis through TRPM7 suppression and promotion of the PI3K/Akt pathway [225,226]. Celastrol (Figure 3C and Table 1), showed its neuroprotection in ischemic stroke by inhibiting the JNK/NF-κB pathway to suppress the inflammatory cascade in the ischemic brain [227]. Rotenone reduces apoptosis in a model of Parkinson’s disease by preventing the increase in ROS and the loss of the mitochondrial membrane potential [228].

### 6.4. Other Compounds

Gallic acid and caffeic acid are phenolic acids with reported neuroprotective effects [229,230,231]. Gallic acid restores mitochondrial dysfunction [232] and improves the outcome of post-stroke depression treatment [233]. Caffeic acid improves neurological dysfunction and decreases infarct volume after focal cerebral ischemia in rats, with the inhibition of NF-κBp65 expression and reduction in malondialdehyde content through the downregulation of 5-lipoxygenase [234].

**Table 1:** Classification of anti-SARS-CoV-2 and neuroprotective compounds from natural sources.

Type	Compound	Source	Anti-SARS-CoV-2 Effect	Neuroprotective Activity	Analysis	References
Flavonoids	Baicalein	*Scutellaria* *baicalensis*	Antiviral activity in vitro (EC50: 4.5 μM) Inhibits Mpro, RdRp, and NSP14 in vitro. Reduces viral load and lung damage in infected mice.	Neuroprotective against AD, PD, cerebral ischemia, epilepsy, aging, and cognitive deficits.	In silico In vitro In vivo Clinical trial	[162,235,236,237]
	Luteolin	*Capsicum annuum*	Antiviral activity in vitro (IC_50_: 4.6 μM). Binding to ACE2 and in vitro inhibition of RdRp enzyme.	Suppresses neuroinflammation, microglia and astrocyte activation, and oxidative stress.	In silico In vitro	[186,238,239]
	Hesperidin	*Citrus aurantium*	Inhibits Mpro, PLpro, and RBD-ACE2 binding (100 μM). Blocks the cellular entry of pseudo-particles of SARS-CoV2.	Protects against apoptosis, oxidative stress, and inflammation in AD and PD models. Prevents brain damage.	In silico In vitro Clinical trial	[240,241,242,243,244,245,246]
	Quercetin	*Ginkgo biloba*	Binding to Mpro (Km: 11 μM) and RBD.	Decreases oxidative stress, neuroinflammation, and neurodegeneration.	In silico In vitro Clinical trial	[172,247,248,249,250,251,252]
Alkaloids	Piperine	*Piper longum*	Possible inhibitor of viral proteases. Combination with curcumin promotes symptomatic recovery in COVID-19 patients.	Decreases inflammatory markers IL-1β, TNF-α, and reduces apoptosis.	In silico Clinical trial	[253,254,255,256]
	Berberine	*Tinospora* *cordifolia*	Antiviral activity in vitro (EC_50_: 9.1 μM). Inhibits Mpro and Nsp15. Reduces the inflammation associated with viral replication in the lungs.	Anti-inflammatory, anti-apoptotic, anti-cholinesterase, and anti-amyloid activities. Protects against subarachnoid hemorrhage by inhibiting the HMGB1/NF-κB pathway.	In silico In vitro Clinical trial	[205,213,219,220,257,258]
	Tetrandrine	*Stephaniae tetrandrae*	Antiviral activity in vitro (IC_50_: 284 nM). Binding to Two-Pore Channels (TPCs) affecting the viral endosomal entry pathway.	Reduces neuroinflammation and apoptosis. Neuroprotection in vascular dementia.	In vitro Clinical trial	[259,260,261,262,263,264,265]
Terpenoids	Glycyrrhizic acid	*Glycyrrhiza* *glabra*	Antiviral activity in vitro (EC_50_: 0.44 mg/mL). Binding to NSP-15 and Mpro inhibition during viral replication in vitro. Inhibition of viral replication in one patient.	HMGB1 inhibitor. Prevents neuroinflammation, epileptogenesis, and cognitive impairment	In vitro Clinical trial	[266,267,268,269,270]
	Celastrol	*Tripterygium* *wilfordii* Hook F	Antiviral activity in vitro (EC_50_: 2.34 nM). Binding to Mpro and RBD. Inhibits viral replication and decreases IL-6 in vitro.	Prevents oxidative stress and inflammation in models of cerebral ischemia, AD, and PD.	In vitro	[271,272,273,274]

AD: Alzheimer’s disease; PD: Parkinson’s disease; HMGB1: high mobility group box 1 protein.

Resveratrol, a stilbenoid widely used as an antioxidant, has neuroprotective activity in ischemic stroke [275,276]. Such an effect might be related to the activation of AMPK and the NAD + dependent deacetylase SIRT1, which participates in the adaptation to conditions of energy depletion [277].

Curcumin, the major polyphenolic compound extracted from *Curcuma longa* plants, is an herbal medicine with antitumor, anti-inflammatory, immunomodulatory, antioxidant, antimicrobial, and antiviral activities. Using in vitro models, the antiviral/anti-inflammatory properties of curcumin against SARS-CoV-2 have been evaluated in peripheral blood mononuclear cells (PBMCs), showing an antiviral effect against the DG614 strain and Delta variant. It was also found that pro-inflammatory cytokines (IL-1β, IL-6, and IL-8) released by PBMCs decrease after treatment with curcumin. The results suggest that curcumin affects the replication cycle of SARS-CoV-2 replicative cycle and exhibits virucidal activity with a variant/strain-independent antiviral effect and immunomodulatory properties [278]. 

Curcumin has several desirable properties as a neuroprotective drug, including anti-inflammatory, antioxidant, and anti-protein aggregation activities, with potential for the prevention of neurological diseases such as AD, PD, Huntington’s, head trauma, aging, and stroke [279]. Curcumin decreases the production of inflammatory mediators such as cytokines, chemokines, and adhesion molecules in the brain of cerebral ischemic patients [280]. Since anti-inflammatory molecules are employed to protect COVID-19 patients from neurological disorders and severe organ-level damage, curcumin treatment could play an important role as a neuroprotector.

Although the bioactive component of turmeric derived from curcuma has a variety of pharmacological activities, its use has been limited by its low solubility, poor bioavailability, rapid metabolism, physicochemical instability, and poor pharmacokinetics [281]. However, the encapsulation of curcumin into nanoformulations has been used to improve its pharmacokinetics, systemic bioavailability, and biological activity. Many nanoformulations have been approved for therapeutic use following the conclusion of preclinical and human clinical trials [282].

## 7. Conclusions and Perspectives

At present there are multiple therapeutic alternatives for COVID-19 treatment. However, the precise effects of those therapies in the neurological effects of the disease are unclear or have not been studied yet, especially in the long-term onset. The evidence discussed in this review shows that molecules of natural origin with antioxidant, anti-inflammatory and/or cytoprotective activities reduce neuronal damage and improve cognitive function. Thus, those molecules are attractive candidates to be further studied in the management of COVID-19 patients with neurological symptoms. Importantly, the molecules discussed here have key characteristics that support clinical analysis: (i) they are cheap and easily accessible; (ii) they have good safety profiles and biodistribution to the CNS; and (iii) they have shown efficacy in models of other neurodegenerative diseases, in some cases, validating their use in traditional medicine. Still, there are multiple areas of opportunity in the field. For example, clinical trials designed to test the efficacy of neuroprotective compounds in COVID-19 patients are required. In those studies, pharmacokinetic analysis should include evaluation of the bioavailability to show that concentrations effective for neuroprotection are reached after oral administration. However, those trials may be difficult to run given that worldwide vaccination has reduced the number of patients with relevant clinical pictures. 

A subset of neuroprotective compounds also inhibits SARS-CoV-2 replication or virus–host interaction. Studies analyzing the structure–activity relationship would allow the design of new molecules with selective or enhanced activities and potential clinical translation. Molecules showing anti-SARS-CoV-2 and neuroprotective activities may improve the prevention and/or mitigation of damage to the CNS induced directly or indirectly by SARS-CoV-2, offering additional benefits to COVID-19 patients. 

## Abbreviations

6-hydroxydopamine (6-OHDA); Acute kidney injury: AKI; Alzheimer’s disease: AD; Amyloid beta: Aβ; angiotensin II: Ang II; angiotensin-converting enzyme 2: ACE2; β-secretase 1: BACE-1; blood–brain barrier: BBB; central nervous system: catalase: CAT;CNS; cerebral venous sinus thrombosis: CVT; Coronavirus: CoV; diffuse alveolar damage: DAD; glutathione: GSH; Huntington’s disease: HD; inducible nitric oxide synthase: iNOS; interleukin-1β: IL-1β; Middle East respiratory syndrome: MERS; multiple sclerosis: MS; N-methyl-D-aspartate: NMDA; nonstructural proteins: NSPs; nuclear factor kappa B: NF-kB; olfactory sensory neurons: OSNs; open reading frame: ORF; Parkinson’s disease: PD; peripheral blood mononuclear cells: PBMCs; peripheral nervous system: PNS; polyproteins: pp; reactive oxygen species: ROS; receptor-binding domain: RBD; renin-angiotensin-aldosterone system: RAAS; RNA-dependent RNA polymerase: RdRp; Severe Acute Respiratory Syndrome: SARS; superoxide dismutase: SOD; synaptic vesicle transporter 2A: SV2A; transmembrane protease serine 2: TMPRSS2; variants of concern: VOC; variants of interest: VOI; von Willebrand factor: VWF.
